# Organizing pneumonia secondary to pulmonary cryptococcosis in immunocompromised patient

**DOI:** 10.1016/j.rmcr.2023.101975

**Published:** 2023-12-29

**Authors:** Zentaro Saito, Takanori Ito, Takuma Imakita, Issei Oi, Osamu Kanai, Kohei Fujita

**Affiliations:** a Division of Respiratory Medicine, Center for Respiratory Diseases, National Hospital Organization Kyoto Medical Center, Kyoto, Japan; b Department of Infectious Diseases, National Hospital Organization Kyoto Medical Center, Kyoto, Japan

**Keywords:** Cryptococcosis, *Cryptococcus neoformans*, Organizing pneumonia, Immunocompromised host, Diabetes mellitus, Fungal disease

## Abstract

Pulmonary cryptococcal infections are fungal infections that often occur in immunocompromised patients and present with a variety of radiographic patterns ranging from nodular to infiltrative shadows. In the present case, we experienced a rare case of organizing pneumonia due to cryptococcal infection in a 71-year-old woman with rheumatoid arthritis. Transbronchial lung biopsy showing fibrotic changes in the alveolar walls, small granulation lesions and cryptococcal organisms with positive Grocott staining. Serum cryptococcal antigen was also found to be positive. Based on these findings, we confirmed the diagnosis of secondary organizing pneumonia due to cryptococcal infection. Treatment with corticosteroids and antifungal drugs led to improvement of the cough and reduction of organizing pneumonia. In immunocompetent patients with organizing pneumonia, it is essential to perform bronchoscopic lung biopsies and serum antigen tests to search for the cause, whenever possible, as it may be due to an infection caused by Cryptococcus, as in the present case.

## 1 Introduction

Pulmonary cryptococcosis is a fungal infection caused by *Cryptococcus neoformans*, which is found in soil and grows in the droppings of pigeons and other birds [1]. Delayed diagnosis and treatment can lead to infection of the central nervous system and a potentially fatal course [1]. Pulmonary cryptococcosis in immunocompetent patients is often asymptomatic, whereas fever and cough are relatively common in immunocompromised patients [2,3]. The most common radiological finding is a nodular shadow in immunocompetent patients, but cryptococcosis in immunocompromised patients shows a variety of radiological findings, including infiltrative shadows besides the nodular shadows [2,[4], [5], [6]].

Cryptococcosis infection as a cause of organizing pneumonia is not well known and only a few cases have been reported so far [7,8]. We herein report a case of pulmonary cryptococcosis in an immunocompromised patient with radiological findings and pathological organizing pneumonia.

## 2 Case presentation

**Fig. 1:**
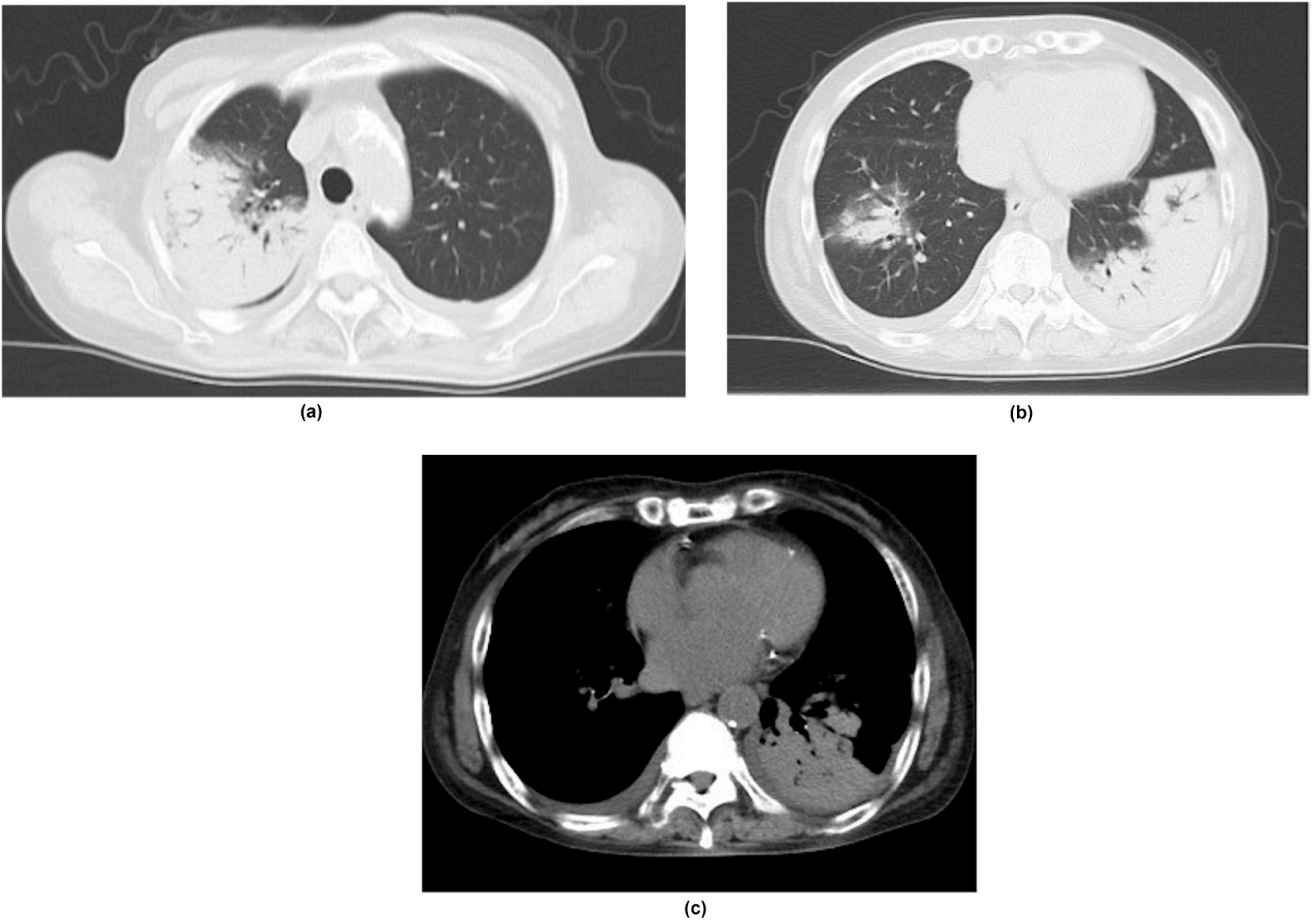
Chest CT at first examination. Chest CT showed multiple infiltrating shadows in the right upper and lower lobes and the left lower lobe. A small amount of pleural effusion was observed in both lung fields.

**Fig. 2:**
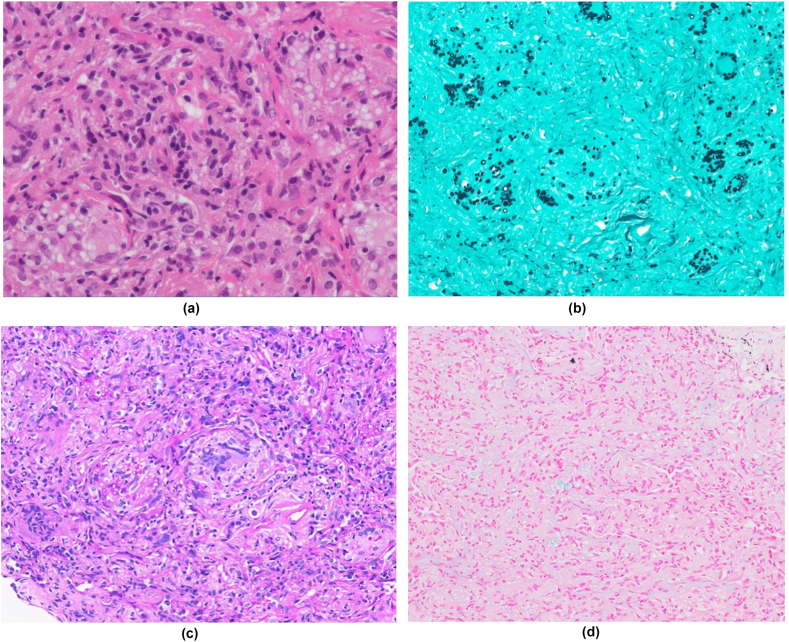
Histological specimens from transbronchial lung biopsies. Granuloma formation with multinucleated giant cells and phagocytosing yeast-like fungi, and spherical fungi of different sizes were observed. (A) Hematoxylin and eosin stain, × 400 (B) Grocott stain, × 100 (C) Periodic acid-Schiff stain, × 100 (D) Alcian blue stain, × 100. (For interpretation of the references to colour in this figure legend, the reader is referred to the Web version of this article.)

A 71-year-old Japanese woman with type 1 diabetes and taking methotrexate (8 mg weekly) for rheumatoid arthritis came to our hospital with a cough and dyspnea. She had no history of allergies or smoking, but she had kept parakeets for five years and had a shrine near her home. There was no apparent contact with pigeons. Chest CT scan showed multiple infiltrative shadows in both lung fields and a small amount of bilateral pleural effusions (Fig. 1). Laboratory testing revealed a white blood cell count of 11,000/mm^3^ with 85 % neutrophils, C-reactive protein of 4.5mg/dl, and positive for cryptococcal antigen test. Tumor markers, (1,3)-β-D glucan and KL-6 were within normal limits. No abnormal cardiac function was observed. Since suspecting idiopathic organizing pneumonia, lung cancer, rheumatoid arthritis-related lung disease, methotrexate-related lymphoproliferative disease, and lung infection, a transbronchial lung biopsy was performed to confirm the diagnosis. The biopsy specimen revealed fibrotic changes in the alveolar wall and numerous small granulomatous species. No Masson bodies were found in the alveolar space. It was also positive for periodic acid schiff (PAS) staining, Grocott and Alcian blue staining, and round to oval structures consistent with cryptococcosis was identified in the alveoli (Fig. 2). No malignant evidence, including lymphoma, was observed. Bronchoalveolar lavage fluid (BALF) examination revealed a cell population of 5.0 % lymphocytes, 5.0 % neutrophils, and 90 % alveolar macrophages, with no pathogens detected in the culture. To rule out an intracranial lesion, a lumbar puncture was performed. The specimen obtained by lumbar puncture was negative for the cryptococcal antigen test, India ink stain, and fungal culture. Based on these results, the patient was diagnosed with organizing pneumonia secondary to pulmonary cryptococcal infection.

Because this patient had a strong cough and dyspnea on admission and was suspected to have organizing pneumonia on chest CT scan imaging, corticosteroid treatment was started before the diagnosis of cryptococcosis. After one week of prednisolone 30 mg, the infiltrative shadows in both lung fields on chest CT scan were reduced and cough and dyspnea were no longer seen. After the diagnosis of pulmonary cryptococcosis, liposomal amphotericin B (L-AMB) (100 mg daily) was continued until cryptococcal meningitis could be ruled out. After confirmation of negative spinal fluid culture, the patient was switched to fluconazole (400 mg daily).

**Fig. 3:**
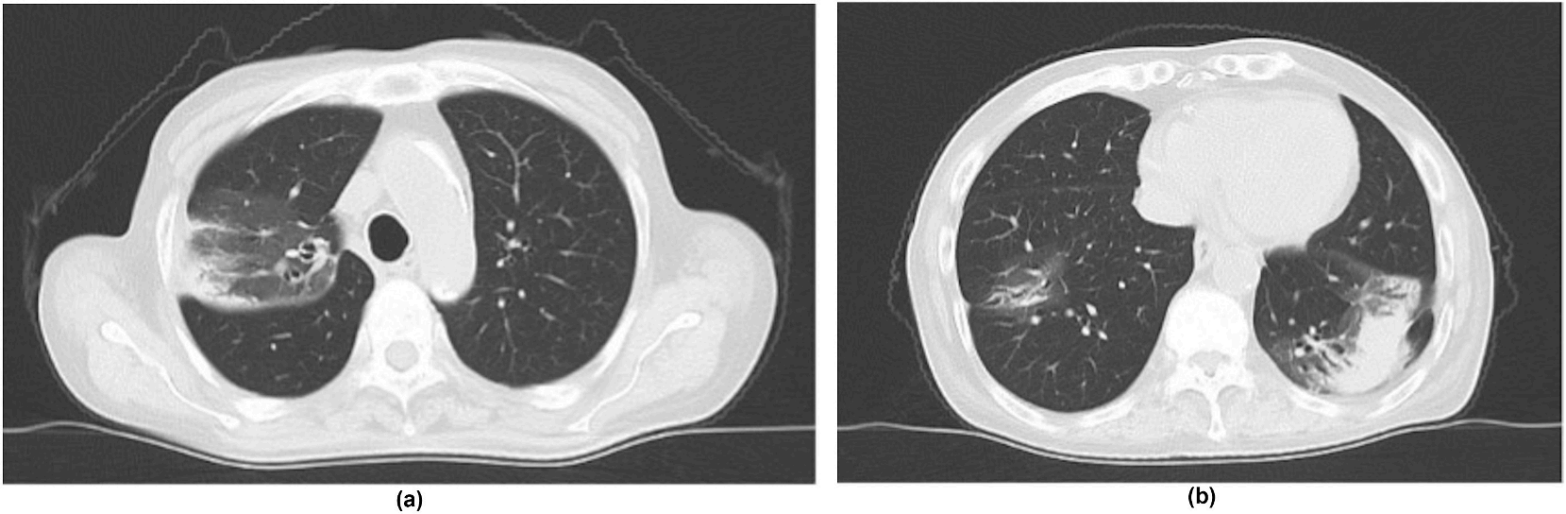
Chest CT scan 2 months after initiating corticosteroids and antifungal drugs. The infiltration shadows in both lung fields were reduced and pleural effusions disappeared.

The patient developed drug-induced renal impairment due to L-AMB, which improved with the drug switch. Two months after starting antifungal medication, a CT scan showed that the infiltrate shadow had shrunk, and the pleural effusion had disappeared (Fig. 3). Owing to the clinical improvement in pulmonary cryptococcosis and the absence of adverse events with antifungal therapy, we intend to treat the patient for 6 months in accordance with guidelines. In addition, prednisolone will be tapered and off in about 3 months.

## 3 Discussion

We experienced secondary organizing pneumonia associated with pulmonary cryptococcosis. In this case, biopsy tissue was positive for PAS, Grocott and Alcian blue staining and pathologically proved cryptococcosis. Also, the pathology showed fibrosis of the interstitium and phagocytosis of macrophages in the alveolar space, and improvement of cough and shortness of breath and reduction of infiltrative shadows on chest CT images after monotherapy with corticosteroids, which led to the diagnosis of organizing pneumonia. Unfortunately, the cell population of the bronchoalveolar lavage fluid did not demonstrate lymphocyte predominance, but this may have been due to the one-week treatment with prednisolone 30 mg before bronchoscopy.

Primary cryptococcosis often shows nodular shadows, but pulmonary cryptococcosis in an immunocompromised state, as in the present case, is known to show an organizing pneumonia pattern on CT images [5,9]. A previous report showed that 30 % of pulmonary cryptococcosis with rheumatoid arthritis exhibited infiltrative shadows [9]. The patient also had bilateral pleural effusions on the first examination. Cardiogenic pleural effusion was not compatible because cardiac function was normal and there was no elevation of BNP. Pleural effusions caused by cryptococcosis are rare but have been reported [10,11]. In the present case, the pleural effusion resolved after antifungal treatment was started, suggesting that it was due to cryptococcosis.

Organizing pneumonia can be caused by a variety of reasons, including idiopathic, collagenous, infectious, malignant and drug-induced [12]. However, organizing pneumonia caused by infectious diseases accounts for only 9.2 % of cases [13]. Corticosteroid therapy is effective in idiopathic, collagenous and drug-induced cases, but conversely, may worsen the disease in cases of organizing pneumonia caused by infections such as cryptococcosis [14], indicating that treatment of the underlying disease with anti-fungal agents should be given priority [15]. In this case, the bronchoscopy confirmed the diagnosis of cryptococcosis, and antifungal treatment could be given promptly.

To conclude, in immunocompromised patients presenting with organizing pneumonia on radiological findings, it is crucial to consider the possibility of infection, including cryptococcal infection, and to perform bronchoscopy and serum cryptococcal antigen testing.

## Author’s contributions

Zentaro Saito, Takanori Ito and Kohei Fujita cared for the patient. Takuma Imakita, Issei Oi and Osamu Kanai supported and offered advice for patient care. Zentaro Saito drafted the manuscript. Kohei Fujita revised the manuscript. All authors approved the manuscript for submission.

## Declaration of competing interest

There are none to declare.
